# Inhibition of type I interferon induction and signalling by mosquito‐borne flaviviruses

**DOI:** 10.1111/cmi.12737

**Published:** 2017-03-22

**Authors:** Stephanie L. Cumberworth, Jordan J. Clark, Alain Kohl, Claire L. Donald

**Affiliations:** ^1^ MRC‐University of Glasgow Centre for Virus ResearchGlasgowScotlandUK

**Keywords:** *Flavivirus*, interferon antagonists, sfRNA, type I interferon

## Abstract

The *Flavivirus* genus (*Flaviviridae* family) contains a number of important human pathogens, including dengue and Zika viruses, which have the potential to cause severe disease. In order to efficiently establish a productive infection in mammalian cells, flaviviruses have developed key strategies to counteract host immune defences, including the type I interferon response. They employ different mechanisms to control interferon signal transduction and effector pathways, and key research generated over the past couple of decades has uncovered new insights into their abilities to actively decrease interferon antiviral activity. Given the lack of antivirals or prophylactic treatments for many flaviviral infections, it is important to fully understand how these viruses affect cellular processes to influence pathogenesis and disease outcome. This review will discuss the strategies mosquito‐borne flaviviruses have evolved to antagonise type I interferon mediated immune responses.

## 1 INTRODUCTION

The *Flavivirus* genus (*Flaviviridae*) encompasses a myriad of viruses transmitted by blood‐feeding arthropod species, several of which represent emergent or re‐emergent pathogens. Important examples include Zika (ZIKV), dengue (DENV), yellow fever (YFV), Japanese encephalitis (JEV), and West Nile (WNV) viruses. Human flavivirus infections are responsible for significant morbidity and mortality worldwide, eliciting a spectrum of manifestations: from asymptomatic infections to mild flu‐like symptoms, or more severe complications such as encephalitis and haemorrhagic fever. Furthermore, congenital developmental deficits, and neurological syndromes have been associated with ZIKV infections, a previously neglected member of the genus (Cao‐Lormeau et al., 2016; de Oliveira & Da Costa Vasconcelos, 2016; Fauci & Morens, 2016; Gould & Solomon, 2008; Mackenzie, Gubler, & Petersen, 2004; Oehler et al., 2014; Ventura, Maia, Bravo‐Filho, Gois, & Belfort, 2016).

**Figure 1:**
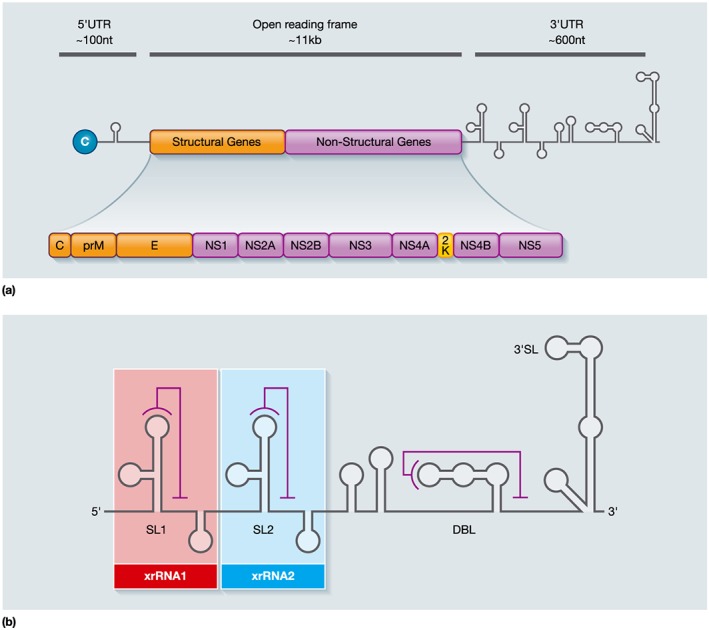
(a) Organisation of the flavivirus genome. The flavivirus genome is composed of a single‐stranded, positive‐sense RNA, of approximately 11 kb. The single open reading frame contains the three structural proteins (C: capsid, prM: premembrane, E: envelope) and seven nonstructural (NS) proteins (NS1, NS2A, NS2B, NS3, NS4A, NS4B, and NS5). These are flanked on either side by highly structured 5′ and 3′ untranslated regions. The gene products are generated from the single polyprotein by co‐ and posttranslational cleavage. This also results in the production of the 2K peptide between NS4A and NS4B. (b) Structure of ZIKV subgenomic flavivirus RNA (sfRNA), as predicted following structural studies and RNA folding analysis. Although the structure of sfRNA varies for different flaviviruses, they all contain similar motifs. All flavivirus sfRNAs contain stem loop (SL) and dumbbell (DBL) structures, which consist of conserved nucleotides capable of forming pseudoknots (PK). PK are represented by lines. Two sfRNAs of differing size are produced during ZIKV infection due to the stalling of XRN1 at the SL structures. Predicted sfRNAs: stalling at SL1 produces xrRNA1 (red box), and xrRNA2 (blue box) is produced by stalling at SL2 (Akiyama et al., 2016; Donald et al., 2016)

Flaviviruses are enveloped viruses and possess an 11 kb single stranded, positive sense RNA genome, encoding a single open reading frame flanked by highly structured 5′ and 3′ untranslated regions (UTRs; Lindenbach, Murray, Thiel, & Rice, 2013). During infection, the viral polyprotein is processed to yield three structural (C: capsid, prM: premembrane, E: envelope) and seven nonstructural proteins (NS1, NS2A, NS2B, NS3, NS4A, NS4B, and NS5; Figure 1a). In addition, all flaviviruses investigated have been shown to produce subgenomic flavivirus RNA (sfRNA), a nongene product generated from incomplete degradation of genomic RNA by the 5′‐3′ exoribonuclease, XRN1 (Clarke, Roby, Slonchak, & Khromykh, 2015; Donald et al., 2016; Pijlman et al., 2008; Roby, Pijlman, Wilusz, & Khromykh, 2014).

Type I interferon (IFN‐I) is crucial in the fight against virus infections. Upon activation, the host's IFN‐I response establishes an antiviral state within the target cell and signals to neighbouring cells. In order to mount a successful innate immune response, eukaryotic organisms must first be able to detect the invading pathogen. This is achieved through the use of a variety of receptors, known as pathogen recognition receptors (PRRs), which are located on both the cell surface and within the cytoplasm. These receptors detect peptides or nucleotides derived from the pathogen, which are known as pathogen associated molecular patterns (PAMPs). There are several families of PRRs, but the most important for flavivirus infections are Toll‐like receptors (TLRs) and RIG‐I like receptors (RLRs) (Munoz‐Jordan & Fredericksen, 2010; Suthar, Aguirre, & Fernandez‐Sesma, 2013). TLRs are membrane bound and, in humans, the TLR family contains 10 members, each of which detects specific PAMPs. Of importance during flavivirus infections are TLR7 and TLR8, which identify single‐stranded RNA (ssRNA), as well as TLR3, which detects double stranded RNA (dsRNA) produced during viral replication. As most viruses produce dsRNA during replication, TLR3 is triggered during the majority of infections. With the exception of TLR3, all TLRs signal through an intermediate protein, MyD88, which eventually leads to activation of the NF‐ĸb, MAPK, ERK, and JNK pathways. Conversely, TLR3 signals through a MyD88 independent pathway, which results in the recruitment of TRIF. This then signals through the TRAF3 and RIP1 signalling pathways to activate the transcription factors IFN‐regulatory factor (IRF)‐3, NF‐ĸB, and AP‐1 to stimulate the IFN‐I pathway (Uematsu & Akira, 2007).

Also involved in the detection of cytoplasmic dsRNA are the RLRs: RIG‐I and Melanoma Differentiation‐Associated protein 5 (MDA5) (Kato et al., 2006). RIG‐I binds to the 5′‐phosphorylated ends of dsRNA molecules, whereas MDA5 binds internally. Both contain a DExD/Hbox helicase domain and a C‐terminal domain, which are involved in the binding of viral dsRNA. In addition, they possess tandem N‐terminal caspase recruitment domains (CARDs), which interact with mitochondrial antiviral‐signalling protein (MAVS), the intermediate signalling molecule located on the outer membrane of mitochondria. This then signals through IRF3/7 to activate the transcription of I IFNs (Gack, 2014; Reikine, Nguyen, & Modis, 2014).

I IFNs bind to the IFN‐α receptor (IFNAR), a heterodimeric transmembrane receptor consisting of two subunits, IFNAR1 and IFNAR2. This results in the recruitment and activation of tyrosine kinases, Janus kinase (JAK1), and tyrosine kinase 2 (Tyk2), through auto‐ and trans‐ phosphorylation. These recruit and phosphorylate the cytoplasmic transcription factors, signal transducer and activation of transcription (STAT) 1 and 2. STAT2 is activated by Tyk2, which is proceeded by the recruitment and phosphorylation of STAT1 by JAK1. The activated STAT1/2 proteins heterodimerise, translocate to the nucleus and associate with IRF‐9 to form the interferon‐stimulated gene factor 3 (ISGF3) complex. ISGF3 binds to the IFN‐stimulated response element (ISRE), which directly induces an antiviral state through the production of several hundred IFN stimulation genes (ISGs) (Ivashkiv & Donlin, 2014; Schneider, Chevillotte, & Rice, 2014; Schoggins et al., 2011).

Recent findings have also suggested that in addition to RIG‐I/MAVS and IFN‐I signalling pathways, the cGAS‐STING pathway is involved in restricting flavivirus infections (Gack & Diamond, 2016; Ma & Damania, 2016). Known to be involved in the detection of DNA viruses, it exhibits activity against particular positive sense RNA viruses which do not involve DNA intermediates as part of their life cycle. Studies involving WNV have illustrated that cGAS (cyclic GMP‐AMP synthase) knockout mice were more susceptible to infection and suggested that in the absence of cGAS, base levels of certain antiviral ISGs are reduced, causing the cell to be more permissive to infection (Schoggins et al., 2011; Schoggins et al., 2014). Similarly, silencing of stimulator of IFN genes (STING) resulted in enhanced DENV replication due to a decrease in the induction of proinflammatory cytokines (Aguirre et al., 2012; Yu et al., 2012). The importance of the role of the cGAS‐STING pathway in RNA virus restriction is illustrated by the inhibitory function of different viral proteins to prevent pathway activation as both DENV and YFV inhibit the activity of STING through interactions with NS2B‐NS3 and NS4B, respectively (Aguirre et al., 2012; Ishikawa, Ma, & Barber, 2009; Yu et al., 2012).

To facilitate propagation, viruses have evolved mechanisms to subvert host responses such as those mediated by IFN‐I (Randall & Goodbourn, 2008; Versteeg & Garcia‐Sastre, 2010). Similarly, flaviviruses have developed several strategies involving one or more of their nonstructural proteins, in addition to sfRNA, as specific IFN‐I antagonists to surmount these host immune responses; although, the viral effectors and mechanisms may differ between viruses (Table 1). It is important to recognise the factors, which underlie these immune evasion strategies in order to understand how they impact disease pathogenesis and for focused vaccine development. Herein, we review select flavivirus encoded products and their IFN‐I antagonist capabilities.

**Table 1:** Summary of type 1 interferon inhibitory activities of flaviviral nonstructural proteins and sfRNA.

Interferon antagonist	Virus	Activity
NS2A	DENV	Inhibition of the JAK/STAT signalling pathway by decreasing STAT1 phosphorylation
	KUNV	Suppression of IFN‐β transcription
NS4B	DENV	Completely blocks interferon signalling (in combination with NS2A and NS4A)
	DENV, YFV, WNV	Inhibition of the JAK/STAT signalling pathway by decreasing STAT1 phosphorylation
	YFV	Interacts with STING to block RIG‐I stimulation
NS2B‐NS3	DENV	Cleaves MITA or STING
		Inhibits IFN production by interacting directly with IκB kinase ε, disrupting RIG‐I signalling, blocking serine 386 phosphorylation, and inhibiting IRF3 nuclear translocation

NS5	DENV	Targets STAT2 for ubiquitin mediated proteasomal degradation involving interactions with UBR4
	ZIKV	Induces ubiquitin mediated proteasomal degradation of STAT2
	YFV	Binds and inhibits STAT2 following IFN‐I induced phosphorylation of STAT1, requires K6 ubiquitination
	WNV	Inhibits STAT1 phosphorylation
	JEV	Blocks Tyk2 phosphorylation
sfRNA	DENV‐2	Sequesters G3BP1, G3BP2, and CAPRIN1,
		Binds and inhibits TRIM25
	ZIKV	Inhibits IFN‐I response downstream of RIG‐I & MDA5
	WNV	Inhibits IFN‐I response through unknown mechanism
	JEV	Inhibits IRF‐3 phosphorylation and nuclear localisation

*Note*. DENV = dengue virus; IFN = interferon; IFN‐I = type I interferon; IRF = IFN‐regulatory factor; JAK = Janus kinase; JEV = Japanese encephalitis virus; KUNV = Kunjin virus; NS = nonstructural; sfRNA = subgenomic flavivirus RNA; STAT = signal transducer and activation of transcription; STING = stimulator of the IFN genes; TRIM = tripartite motif‐containing protein; Tyk2 = tyrosine kinase 2; UBR4 = ; Ubiquitin protein ligase E3 component N‐Recognin 4; WNV = West Nile virus; YFV = yellow fever virus; ZIKV = Zika virus.

### 1.1 NS2A

The flavivirus NS2A protein is small (20 kD), hydrophobic, and associated with the endoplasmic reticulum. It is a multifunctional protein with roles in virion assembly (Kummerer & Rice, 2002; Leung et al., 2008), RNA replication (Mackenzie, Khromykh, Jones, & Westaway, 1998; Rossi, Fayzulin, Dewsbury, Bourne, & Mason, 2007), membrane permeabilisation (Chang et al., 1999), and dissemination from infected mosquito midguts (Mcelroy, Tsetsarkin, Vanlandingham, & Higgs, 2006). It has also been shown to act as an interferon antagonist, which has been described for WNV (Liu et al., 2006), Kunjin virus (KUNV, a WNV variant) (Liu, Chen, Wang, Huang, & Khromykh, 2004; Liu et al., 2005), and DENV‐2 (Munoz‐Jordan, Sanchez‐Burgos, Laurent‐Rolle, & Garcia‐Sastre, 2003). During DENV infection, it is known to reduce IFN‐α/β signalling through inhibition of the JAK/STAT signalling pathway to impede the induction of ISGs. Individual expression of NS2A, as well as NS4B and NS4A, facilitated the replication of an IFN‐sensitive virus, GFP‐tagged Newcastle disease virus (NDV‐GFP), with NS4B being the most potent. The combined action of DENV‐2 NS2A along with NS4A and NS4B was sufficient to block IFN signalling completely through a reduction in STAT1 phosphorylation, prohibiting its nuclear localisation and preventing IFN‐β promoter driven transcription from two ISREs (Munoz‐Jordan et al., 2003). Research on KUNV has shown that a single amino acid substitution (A30P) is responsible for the suppression of IFN‐β transcription both *in vitro* and *in vivo*, and results in diminished virulence in mice (Liu et al., 2004; Liu et al., 2006; Melian et al., 2013). Infection by viruses containing this mutation are highly attenuated, and the production of IFN‐I is swift and continuous, allowing them to establish a productive infection in IFN competent cell lines; however, the exact mechanism and cellular target of its control are unknown (Liu et al., 2006).

### 1.2 NS2B‐NS3

The NS2B protein interacts with NS3 to form a stable complex which functions as a serine protease (Falgout, Pethel, Zhang, & Lai, 1991). Studies have illustrated that the NS2B‐NS3 protease of DENV interferes with IFN‐I induction via cleavage of MITA/STING (Aguirre et al., 2012; Yu et al., 2012). Furthermore, through the direct interaction and modulation of IκB kinase ε, an important kinase involved in IFN‐I induction, DENV NS2B‐NS3 disrupts RIG‐I signaling, blocks serine 386 phosphorylation and nuclear transport of IRF3 thereby decreasing IFN production (Anglero‐Rodriguez, Pantoja, & Sariol, 2014).

### 1.3 NS4B

NS4B is known to be an important IFN‐I signalling antagonist during DENV‐2 infections by inhibiting the JAK/STAT pathway. It functions by antagonising STAT1 phosphorylation and inhibiting its nuclear localisation thus preventing ISG induction. This activity has been documented for both YFV and WNV showing conservation between these mosquito‐borne viruses (Munoz‐Jordan et al., 2003; Munoz‐Jordan et al., 2005). The N‐terminal 2K signal peptide sequence of NS4B (Figure 1a) has also been indicated as critical to IFN inhibition; although, it can be substituted for another signal peptide with no impact on NS4B function (Munoz‐Jordan et al., 2003). The activity of NS4B depends upon its insertion into the ER membrane following NS4A/NS4B cleavage by the NS2B‐NS3 serine protease. Although its specific mechanism has not yet been established, the initial 125 amino acids alone are required for IFN‐I inhibition. In particular, amino acids 77–103 are suggested to interact with cytoplasmic components involved in IFN stimulation and may be important for antagonistic activity (Munoz‐Jordan, 2010; Munoz‐Jordan et al., 2003). Alternatively, in WNV NS4B residues E22 and K24 have been shown to be key to IFN suppression (Munoz‐Jordan et al., 2003). Unlike DENV, YFV NS4B blocks RIG‐I through an interaction with STING (Ishikawa et al., 2009). This highlights strain‐specific variations used for IFN suppression between different flaviviruses.

### 1.4 NS5

NS5 is the largest, most conserved protein amongst flaviviruses. It confers two enzymatic activities via the N‐terminal methyltransferase domain, implicated in producing the viral RNA 5′ cap with N7 and 2′‐O methylation, and the C‐terminal RNA dependant RNA polymerase (RdRp), which replicates viral RNA (Chang et al., 2016; Davidson, 2009). The methyltransferase activity of NS5 offers some protection for the virus by producing capped viral RNA, enabling host RNA mimicry. Methylation at the N7 and 2′‐O sites disguises viral RNA from cytoplasmic PRRs that recognise single‐stranded RNA possessing a terminal 5′ triphosphate—a signature of “foreign” RNA—and prevents identification by IFN‐induced protein with tetratricopeptide repeats 1 (IFIT1) (Chang et al., 2016; Daffis et al., 2010; Decroly, Ferron, Lescar, & Canard, 2011; Jensen & Thomsen, 2012; Kimura et al., 2013; Szretter et al., 2012). In addition to these enzymatic functions, NS5 has been described as a potent flavivirus IFN‐I antagonist (Best, 2017). Despite its highly conserved nature, the mechanisms by which it dampens the IFN‐I response vary substantially; although, STAT inhibition has been described as common mode of action for some flaviviruses.

NS5 inhibition of STAT1/2 activation or translocation prevents the upregulation of ISGs and the establishment of an antiviral state. DENV NS5 binds and degrades STAT2 by targeting it for Ubiquitin‐mediated proteasomal degradation (Ashour, Laurent‐Rolle, Shi, & Garcia‐Sastre, 2009; Mazzon, Jones, Davidson, Chain, & Jacobs, 2009). Ectopic expression of NS5 alone was not sufficient to induce STAT2 degradation. It has been shown that NS5 maturation via N‐terminal cleavage is required for STAT2 depletion, although the role that this plays is unclear (Ashour et al., 2009). Degradation is not dependent on the terminal amino acid residue as both plasmid expressed NS5 with a terminal methionine, as well as NS5 produced during a native infection with a terminal glycine are functional (Ashour et al., 2009). Ubiquitin protein ligase E3 component N‐Recognin 4 (UBR4) has been identified as binding to DENV NS5 and promoting STAT2 degradation. DENV NS5 acts as a bridge between UBR4 and STAT2, but this appears to be specific to DENV and is not seen with YFV or WNV (Morrison et al., 2013). The first 10 amino acids of DENV NS5 are required for UBR4 binding, and threonine and glycine at positions 2 and 3 respectively were identified as critical for UBR4 binding and STAT2 degradation (Ashour et al., 2009; Morrison et al., 2013). These residues are conserved in all DENV serotypes but not in other flaviviruses (Morrison et al., 2013). Furthermore, it was found that the NS5‐UBR4 interaction is independent of STAT2. UBR4 lacks an ubiquitin ligase catalytic domain, and therefore it has been suggested to act as a scaffold for ubiquitination to target STAT2 for proteasomal degradation (Morrison et al., 2013). More recently, ZIKV has also been shown to bind and deplete STAT2 via proteasomal degradation. However, unlike DENV, this is independent of the production of an authentic NS5 N‐terminus and UBR4 interaction (Grant et al., 2016). The interaction between NS5 and STAT2 as well as the suppression of described as a host species specific affect for both ZIKV and DENV. NS5‐STAT2 binding is abolished in mouse model systems possessing intact IFN signalling pathways, and this significantly impedes virus infection (Ashour et al., 2010; Grant et al., 2016). The converse is observed in mice lacking an intact IFN system where infections are lethal. Therefore, virus–host interactions at the level of IFN‐I antagonism have significant implications in the development of suitable infectious model systems.

Similar to DENV, the extreme N‐terminus of YFV also contains a motif required for NS5‐STAT2 interactions and subsequent inhibition (Laurent‐Rolle et al., 2014). Curiously, the YFV NS5‐STAT2 interaction and resulting IFN‐I antagonism is dependent on stimulation with IFN‐I; a mechanism thus far unique to YFV in the flavivirus genus (Laurent‐Rolle et al., 2014). YFV NS5 does not target STAT2 for proteasomal degradation unlike DENV. Instead, IFN‐I induced phosphorylation of STAT1, in addition to K63‐linked polyubiqutination via E3 ligase Tripartite motif‐containing protein 23 (TRIM23) at K6 of NS5, is required to bind STAT2 and prevents ISGF3 interaction with the IRSE promoter (Laurent‐Rolle et al., 2014).

The NS5 of virulent WNV strain NY99 has been shown to be a potent inhibitor of IFN through the inhibition of STAT1 phosphorylation (Laurent‐Rolle et al., 2010). Transient expression of WNV NY99 NS5 alone was sufficient to rescue NDV‐GFP replication in IFN treated cells, whereas expression of KUNV NS5 did not (Laurent‐Rolle et al., 2010). This study was performed in tandem with both virulent and attenuated forms of JEV NS5 protein and suggested the IFN antagonist activity of NS5 appeared to be associated with strain virulence (Laurent‐Rolle et al., 2010). Mutagenesis studies of WNV NY99 demonstrated a single amino acid mutation (F653S) dampens the capability of NS5 to suppress IFN‐β mediated STAT1 phosphorylation and ISRE‐dependent gene expression, whereas the inverse mutation, S653F, in KUNV augments IFN suppression by NS5 (Laurent‐Rolle et al., 2010). This WNV residue, together with W382, VI631/632, and W651, which are also shown to be important in IFN‐I suppression, lies within a structural pocket identified in Langat virus to map to the indispensable RdRp domain (Park, Morris, Hallett, Bloom, & Best, 2007).

The action of JEV NS5 presents an alternative mechanism of IFN‐I signalling inhibition through a Tyk2 phosphorylation blockade. This induces the cytoplasmic retention of STAT1/2 and prevents IRSE driven transcription (Lin, Chang, Yu, Liao, & Lin, 2006). No direct physical association between JEV NS5 and IFN‐I signalling molecules Tyk2, STAT1, or JAK1 has been demonstrated. Instead the use of protein tyrosine phosphatases ablates NS5 mediated inhibition of IFN‐I signalling, suggesting that JEV NS5 may act through cellular tyrosine phosphatases to exert antagonistic affects (Castillo Ramirez & Urcuqui‐Inchima, 2015; Lin et al., 2006).

### 1.5 Flavivirus subgenomic RNA (sfRNA)

Whilst it has been known for over a decade that flavivirus nonstructural proteins play important roles in the evasion and antagonism of the host immune response, the antagonistic properties of sfRNA has more recently came to light. sfRNA is produced during the course of flavivirus infection of vertebrate cells as a result of incomplete digestion of the 3'UTR by the cellular exonuclease, XRN1 (Clarke et al., 2015; Pijlman et al., 2008; Roby et al., 2014). The production of these small RNAs, which are typically around 500 nt, has been shown to be specific to flaviviruses (Akiyama et al., 2016; Donald et al., 2016; Lin, Chang, & Chang, 2004; Liu, Chen, & Khromykh, 2003; Moon et al., 2015; Pijlman et al., 2008; Schnettler et al., 2012; Schnettler et al., 2014; Schuessler et al., 2012).

Work from the Khromykh laboratory, demonstrated the structure and mechanism through which sfRNA is generated (Pijlman et al., 2008). RNA correlating to the relative size of the 3′ UTR was detected in both vertebrate and invertebrate cells infected with various flaviviruses or derivative replicons. Due to the absence of an internal promoter and the apparent reliance on host cell machinery, it was hypothesised that a cellular exoribonuclease may be responsible for its production (Pijlman et al., 2008). This was later shown to be due to stalling by XRN1 (Chapman, Moon, Wilusz, & Kieft, 2014).

The construction of mutant viruses incapable of producing sfRNA demonstrated that the generation of intact sfRNA was necessary for effective viral growth and pathogenicity in cell culture and mice (Pijlman et al., 2008). Whilst the mechanism for this was unclear, sfRNA was proposed to play a modulatory role in the host antiviral response. Indeed, IFN‐β promoter activity was reduced in cells infected with JEV or transfected with JEV‐derived sfRNA (Chang et al., 2013). In these cells, sfRNA inhibited the phosphorylation and nuclear localisation of IRF‐3; although, the mode of action is still to be determined. Furthermore, sfRNA‐deficient WNV and YFV, which replicate poorly in interferon competent cells, are able to replicate successfully in cells deficient in major factors involved in the IFN response (Funk et al., 2010; Schuessler et al., 2012; Silva, Pereira, Dalebout, Spaan, & Bredenbeek, 2010). sfRNA‐deficient WNV was also found to be more sensitive to IFN pretreatment; however, replication was rescued in the presence of INFAR neutralising antibodies. Therefore, sfRNA must interact with the IFN‐I response in infected cells (Schuessler et al., 2012).

During DENV infection, it has been shown that sfRNA antagonises a group of proteins, G3BP1, G3BP2, and CAPRIN1, which have previously been implicated in modulating viral infection through the regulation of several ISGs and ISG mRNA translation (Bidet, Dadlani, & Garcia‐Blanco, 2014; Cobos Jimenez et al., 2015; Humoud et al., 2016; Katsafanas & Moss, 2004). It was also found that DENV‐2 sfRNA colocalises and interacts with G3BP1, G3BP2, and CAPRIN1. A chimeric YFV‐DENV sfRNA that lacked stem loop II (SL‐II) but contained the equivalent YFV structures was shown to have lower binding affinity to G3BP1, and when compared with WT DENV sfRNA, was unable to reduce the transcription of host ISGs. It was suggested that DENV sfRNA sequesters G3BP1, G3BP2, and CAPRIN1, thereby preventing the upregulation of ISG expression. Interestingly, this interaction was not found in experiments using DENV‐3, KUNV, or YFV‐17D 3'UTRs, highlighting that the mechanisms through which sfRNA antagonises the IFN response are highly divergent between other flaviviruses (Bidet et al., 2014). Indeed, ZIKV sfRNA has recently been shown to function as both a RIG‐I and MDA5 agonist and demonstrates broader antagonistic activity compared to DENV‐2, which affects RIG‐I only (Donald et al., 2016).

Structural analysis and RNA‐fold predictions have been used to determine the structure of sfRNAs. Studies mapping the extensive secondary structures of MVEV and DENV sfRNAs revealed particular three‐way helix junction conformations that are required for XRN1 stalling and preservation of the integrity of the RNA (Chapman, Costantino et al., 2014; Chapman, Moon et al., 2014). The crystal structure of MVEV indicates a ring‐like structure in SL‐II, through which the 5′ end of the XRN1‐resistant RNA protrudes. When XRN1 encounters this structure, it attempts to pull the 5′ end of the sfRNA through this ring, causing the structure to tighten and the enzyme to stall (Chapman et al., 2014). In the case of ZIKV sfRNA, it has been determined that two XRN1‐resistant RNAs (xrRNAs) are produced during infection. Referred to as xrRNA1 and xrRNA2, these are produced as a result of XRN1 stalling at SL‐I and SL‐II, respectively (Figure 1b). This differential sfRNA production may be the result of cellular mechanisms; however, the significance of this is unclear (Akiyama et al., 2016). Such data will be very useful for analysing the mechanism of this IFN antagonist further (Akiyama et al., 2016; Donald et al., 2016).

TRIM25, a modulator of the IFN‐I response, has also been identified as a target of DENV sfRNA (Manokaran et al., 2015). TRIM25 functions as an E3 ligase, which adds poly‐ubiquitin chains to the amino‐terminal CARDs of RIG‐I (Gack, 2014). This is thought to facilitate the interaction of RIG‐I with MAVS, thus modulating downstream signalling of the IFN‐I response. TRIM25 and MAVS were also shown to interact with DENV sfRNA; however, although TRIM25 was found to be enriched for bound sfRNA, MAVS was not (Manokaran et al., 2015).

## 2 CONCLUDING REMARKS

The vertebrate IFN response is vital to restrain a number of pathogenic infections, including flavivirus infections. Investigations into flavivirus–host cell interactions have identified a number of important molecular components involved in counteracting this response and contributing to viral pathogenesis and disease development. The evolution of specific IFN‐I response antagonists to subvert the host immune response at definitive stages of the cascade have long reaching effects in terms of viral growth kinetics and fitness, many of which are still to be fully investigated. In particular, enhancing our understanding of sfRNA interactions with cellular immune responses represents an exciting new field of study that may greatly impact our understanding of medically important flavivirus infections. Research has shown that different flaviviruses use different approaches to counteract host innate immune responses, and a better understanding of these interactions is important for the development of effective prophylaxis and anti‐viral therapeutics that will both inhibit the spread of these emerging infections and improve medical outcomes.
